# Safety and effectiveness of indocyanine green fluorescence imaging-guided laparoscopic hepatectomy for hepatic tumor: a systematic review and meta-analysis

**DOI:** 10.3389/fonc.2023.1309593

**Published:** 2024-01-03

**Authors:** Kan Zhou, Shumin Zhou, Lei Du, Erpeng Liu, Hao Dong, Fuping Ma, Yali Sun, Ying Li

**Affiliations:** ^1^ Department of Hepatobiliary Surgery, Xianyang Central Hospital, Xianyang, Shaanxi, China; ^2^ Department of General Surgery, The First Affiliated Hospital of Xi’an Jiaotong University, Xi’an, Shaanxi, China; ^3^ Clinical Medical College, Xi’an Medical University, Xi’an, Shaanxi, China

**Keywords:** indocyanine green, fluorescence imaging, laparoscopic hepatectomy, meta-analysis, systematic review

## Abstract

**Introduction:**

Previous clinical investigations have reported inconsistent findings regarding the feasibility of utilizing indocyanine green fluorescence imaging (ICGFI) in laparoscopic liver tumor removal. This meta-analysis aims to comprehensively evaluate the safety and effectiveness of ICGFI in laparoscopic hepatectomy (LH).

**Methods:**

A systematic search of pertinent clinical studies published before January 30^th^, 2023 was conducted in databases including PubMed, Embase, Cochrane, and Web of Science. The search strategy encompassed key terms such as “indocyanine green fluorescence,” “ICG fluorescence,” “laparoscopic hepatectomy,” “hepatectomies,” “liver Neoplasms,” “hepatic cancer,” and “liver tumor.” Additionally, we scrutinized the reference lists of included articles to identify supplementary studies. we assessed the quality of the incorporated studies and extracted clinical data. Meta-analysis was performed using STATA v.17.0 software. Either a fixed-effects or a random-effects model was employed to compute combined effect sizes, accompanied by 95% confidence intervals (CIs), based on varying levels of heterogeneity.

**Results:**

This meta-analysis encompassed eleven retrospective cohort studies, involving 959 patients in total. Our findings revealed that, in comparison to conventional laparoscopic hepatectomy, patients receiving ICGFI-guided LH exhibited a higher R0 resection rate (OR: 3.96, 95% CI: 1.28, 12.25, *I^2 =^* 0.00%, *P* = 0.778) and a diminished incidence of intraoperative blood transfusion (OR: 0.42, 95% CI: 0.22, 0.81, *I* ^2 =^ 51.1%, P = 0.056). Additionally, they experienced shorter postoperative hospital stays (WMD: −1.07, 95% CI: −2.00, −0.14, *I* ^2 =^ 85.1%, P = 0.000). No statistically significant differences emerged between patients receiving ICGFI-guided LH vs. those undergoing conventional LH in terms of minimal margin width and postoperative complications.

**Conclusion:**

ICGFI-guided LH demonstrates marked superiority over conventional laparoscopic liver tumor resection in achieving R0 resection and reducing intraoperative blood transfusion rates. This technique appears to hold substantial promise. Nonetheless, further studies are needed to explore potential long-term benefits associated with patients undergoing ICGFI-guided LH.

**Systematic review registration:**

https://www.crd.york.ac.uk/prospero/, identifier CRD 42023398195.

## 1 Introduction

Hepatic tumors pose a significant global health challenge due to their high prevalence and mortality rates. Notably, China shoulders nearly half of the worldwide burden of liver cancer (1, 2). Globally, hepatic tumors rank as the third most common cause of cancer-related fatalities, while in China, they stand as the second leading cause (3, 4). The well-established risk factors of liver cancer include chronic infection with hepatitis B virus (HBV) or hepatitis C virus (HCV), heavy alcohol consumption, metabolic diseases (particularly nonalcoholic fatty liver disease), and exposure to dietary toxins such as aflatoxins and aristolochic acid (5, 6). Disturbingly, it is estimated that there will be a worrisome 55.0% increase in new liver cancer cases annually from 2020 to 2040, potentially resulting in 1.3 million liver cancer-related deaths by 2040 (7). Consequently, there is an urgent need to improve the prognosis for individuals afflicted by liver tumors.

Surgical resection remains the mainstay of curative treatment modality for patients with resectable liver cancer (8). Laparoscopic-assisted hepatectomy has emerged as a promising alternative to open hepatectomy, leading to enhanced clinical outcomes (9). Nowadays, laparoscopic hepatectomy (LH) has become the preferred surgical method for both benign and malignant liver conditions (10, 11). However, despite improvements in minimally invasive surgery and related surgical techniques, accurately and swiftly delineating tumor boundaries during LH continues to present a significant challenge.

In recent years, a revolutionary surgical navigation technology called indocyanine green fluorescence imaging (ICGFI) has gained widespread acceptance across various surgical specialties (12, 13). Compared with conventional imaging methods, ICGFI stands out as a potent intraoperative tool (14). It boasts an impressive 80% detection rate for liver lesions following intravenous ICG administration and achieves an astounding 100% accuracy in detecting lesions within 8 mm from the liver’s surface (15). Due to its heightened sensitivity, resolution, and real-time capabilities, it proves invaluable for precisely locating tumors and determining surgical margins in clinical practice (14, 16, 17). Moreover, ICGFI-guided LH has been successfully applied to liver resection surgery (18, 19). However, the clinical implementation of ICGFI-guided LH remains in its nascent stages, and its potential to assist surgeons in accurately delineating tumor boundaries and achieving complete R0 resections during laparoscopy remains uncertain.

Given the current dearth of robust evidence-based medical data, the role of ICGFI-guided LH in clinical practice remains controversial. In this context, the present study was conducted to evaluate its effectiveness and safety.

## 2 Materials and methods

We complied with the Preferred Reporting Items for Systematic Reviews and Meta-Analyses (PRISMA) guidelines (20) to conduct this study. Our search protocol was prospectively registered with PROSPERO (CRD 42023398195).

### 2.1 Search strategy and eligibility criteria

To identify all pertinent studies comparing ICGFI-guided LH with conventional LH for liver cancer, two researchers performed thorough searches across various databases, including PubMed, Embase, Medline, Web of Science, and the Cochrane Library. Our search encompassed studies available up to January 30^th^, 2023, without any language restrictions. However, for non-English articles, an English abstract was required. The formulation of our search terms was derived from an initial literature review and consultations with relevant experts. These search terms encompassed Liver Neoplasms, Hepatic Cancer, Indocyanine Green, Cardio Green, and so on (as detailed in the **Supplementarey Data Sheet 1**). Additionally, the reference lists of all included articles were examined to identify additional eligible studies.

Eligible studies should meet the following inclusion criteria: (1) Confirmed diagnosis of liver disease followed by LH; (2) An experimental group undergoing ICGFI-guided LH, with the control group receiving laparoscopic hepatectomy without the assistance of ICGFI; (3) A sample size exceeding 15 patients; (4) Studies reporting at least one of the outcome measures we investigated; (5) Study designs included randomized controlled trials (RCTs), case-control studies, cross-sectional studies, and cohort studies.

Exclusion criteria were applied as follows: (1) Participants under the age of 18 years; (2) Duplicate publications; (3) Insufficient or unclear reporting of outcome measures; (4) Types of publications such as case reports, conference presentations, reviews, abstracts, expert opinions, animal studies, technical notes, editorials, and letters.

### 2.2 Data collection and quality assessment

Data from eligible studies was independently extracted by two researchers. Any discrepancies that arose between the researchers were settled through discussion or, when necessary, consultation with a third researcher. A wide array of data was recorded, encompassing baseline characteristics (including first author, publication year, country, study design, sample size, age, sex, type of resection, and ICG-related parameters such as concentration, volume, injection site, timing, and imaging system. We developed a list of nine predefined observation indicators based on an initial literature review. Outcome measures encompassed surgery duration, R0 resection, minimal margin width, blood transfusion, postoperative hospitalization, and postoperative complications (including biliary fistula, hepatic failure, incision-related conditions, pleural effusion, and abdominal ascites). Data on these outcomes were collected from each eligible study, and any instances of missing data were noted. When data were incomplete, our team made efforts to contact the corresponding author for clarification. Data were extracted from the included studies using a standardized template developed by the researchers and maintained in Microsoft Excel (Microsoft, Redmond, WA, USA). The accuracy of data extraction was further affirmed through cross-verification between the authors.

In cases of quantitative data where mean and standard deviation (SD) were not provided, we employed an alternative method to estimate these values, relying on the median, range, and sample size if information from the authors was unavailable (21, 22). To gauge the quality of cohort studies, we utilized the Newcastle-Ottawa Scale (NOS) (23), which assigns a maximum score of 9 points to a study. Studies achieving scores of no less than 7 points were rated as having high quality. Two researchers independently assessed the quality of each study, and any disagreements pertaining to article quality were resolved through discussion or, if necessary, consultation with a third author.

### 2.3 Outcomes and statistical analysis

We conducted statistical analyses utilizing the software STATA v.17.0 (Stata Corp., College Station, TX, USA) for statistical analysis. Continuous variables were estimated using the weighted mean difference (WMD), whereas categorical variables were expressed as odds ratio (OR), accompanied by their respective 95% confidence intervals (CIs). Statistical significance was defined as a P value < 0.05. The degree of heterogeneity among effect sizes was gauged using the *I* ^2^ statistic. In instances of non-significant heterogeneity (*I* ^2^ ≤ 50%), we applied a fixed-effects model, while significant heterogeneity (*I* ^2^ > 50%) required the use of a random-effects model. Additionally, sensitivity analyses were conducted to evaluate the potential impact of individual studies on overall results, especially for outcomes demonstrating significant heterogeneity. To assess potential publication bias, we employed a funnel plot. The asymmetry of the funnel plot was evaluated using Egger’s test and Begg’s test for outcomes involving 10 or more studies (24).

## 3 Results

### 3.1 Study identification, quality and baseline characteristics of eligible studies

**Figure 1:**
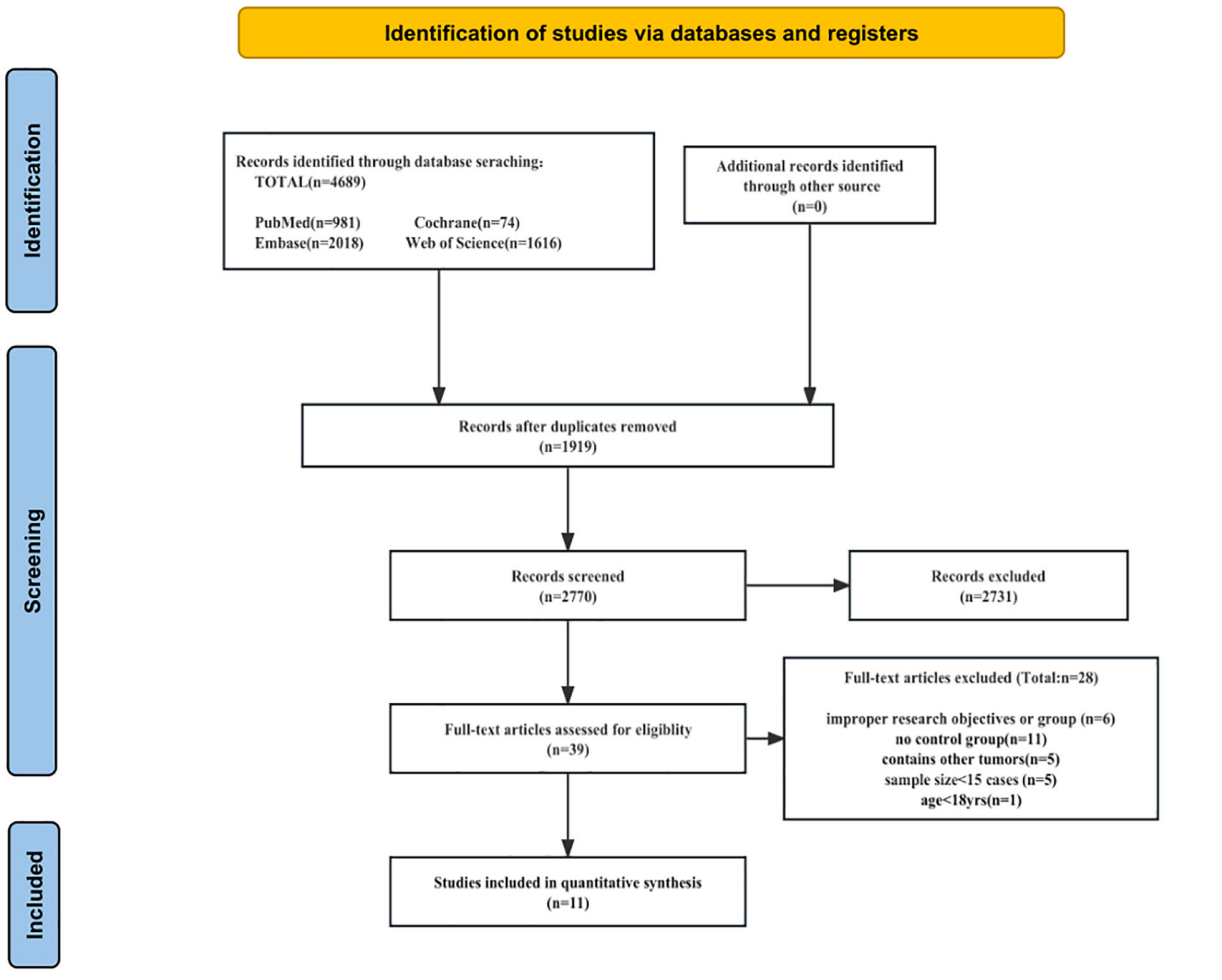
Flow diagram of study selection.

Our approach to conducting the literature search and selection adhered to our predetermined strategy. Initially, we identified a total of 4,689 relevant studies. Among them, 1,919 studies were excluded due to duplication, and an additional 2,731 studies were removed after reviewing their titles and abstracts. The remaining 39 full-text articles were reviewed, followed by the removal of 28 articles for various reasons, resulting in the inclusion of 11 studies (25–35) in the meta-analysis (**Figure 1**). All 11 studies were retrospective cohort studies, with nine conducted in China and the remaining two in Japan. These studies involved 959 patients, with 446 undergoing ICGFI-guided LH and 513 not receiving ICGFI-guided LH. The patient enrollment period for these 11 studies spanned from 2008 to 2021. The cohort studies included in the analysis had NOS scores ranging from 7 to 8, indicating a high level of quality (**Supplementarey Data Sheet 1**). **Table 1** provides a summary of the baseline characteristics of the 11 studies. **Table 2** provides detailed information on the ICG-related indicators reported in 11 studies.

**Table 1:** characteristics of included studies (n=11).

ID	Study	Year	Region	Period	Study design	N (I/C)	Gender (M/F)	Age (years)	Child-pugh (A/B)	HbsAg	cirrhosis	NOS
**1**	Takeshi Aoki	2018	Japan	2008.02-2016.12	Cohort study	25/72	15/44	63(34–84)/69(35–86)	NA	NA	0/0	**8**
**2**	Yu Zhou	2019	China	2017.11-2018.8	Cohort study	21/21	15/15	NA	Grade A (18/18)/Grade B (3/3)	15/15	9/9	**8**
**3**	Peng Zhang	2019	China	2018.01-2018.12	Cohort study	30/34	23/29	55.7 ± 11.2/52.5 ± 12.1	Grade A (27/34)/Grade B (3/0)	17/22	10/11	**7**
**4**	Hao Lu	2020	China	2018.01-2019.12	Cohort study	57/63	38/39	57.3 ± 12.2/55.2 ± 12.5	NA	NA	NA	**8**
**5**	Hao Chen	2022	China	2018.01-2021.10	Cohort study	48/60	43/50	57.3 ± 9.7/56.3 ± 12.1	Grade A (36/46)/Grade B (12/14)	40/41	NA	**7**
**6**	Jian Cheng	2022	China	2019.01-2021.01	Cohort study	24/30	17/19	55.5 ± 12.5/57.9 ± 12.5	Grade A (24/30)/Grade B (0/0)	12/21	8/9	**8**
**7**	Shinji Itoh	2022	Japan	2017.01-2020.12	Cohort study	32/32	20/25	67 (44–83)/69 (44–87)	Grade A (31/32)/Grade B (1/0)	NA	NA	**8**
**8**	Wang Jianxi	2022	China	2014- 2020	Cohort study	81/81	68/69	NA	Grade A (81/81)/Grade B (0/0)	74/73	27/26	**8**
**9**	Fusheng Liu	2022	China	2016.01-2020.12	Cohort study	50/50	46/42	56.82 ± 10.41/59.16 ± 10.82	Grade A (49/48)/Grade B (1/2)	33/34	28/22	**8**
**10**	Yi Zhou	2023	China	2019.1-2021.12	Cohort study	36/36	32/29	56.94 ± 10.23/57.00 ± 10.27	NA	28/29	24/24	**8**
**11**	Zhu Wen	2023	China	2018.06-2021.06	Cohort study	42/34	35/30	52.5 (48.0–65.0)/60.0 (49.8–66.5)	Grade A (40/31)/Grade B (2/3)	NA	25/18	**7**

N(I/C): patients number (intervention group/control group); Gender (M/F): patients number(male/female); Child-pugh (A/B): liver function (grade A/grade B); NOS, Newcastle Ottawa Scale; NA, not applicable.

**Table 2:** The ICG-related indicators of all the studies.

ID	Study	ICG R15 (%)	Intraoperative ICG Dose (mg)	Site of Injection	Imaging System
**1**	Takeshi Aoki	13 (4–17)/16 (3–32)	NA	Peripheral vein	The PINPOINT Endoscopic Fluorescence Imaging System (Novadaq, Mississauga, ON, Canada)
**2**	Yu Zhou	NA	NA	Peripheral vein	The PINPOINT™ imaging system (NOVADAQ, Toronto, Canada).
**3**	Peng Zhang	NA	2.5/2.5	Peripheral veins and portal veins	The white-light HD mode of Pinpoint endoscopic fluorescence imaging system (Novadaq Technologies Inc., Canada)
**4**	Hao Lu	4.2 (2.1–7.4)/3.7 (2.4–6.7)	NA	Peripheral vein	The PINPOINT PC9000 (NOVADAQ, Canada) endoscopic system
**5**	Hao Chen	NA	0.25-0.5/0.75-1.25	Peripheral veins and portal veins	NA
**6**	Jian Cheng	5.8 ± 2.2/6.4 ± 1.7	2.5	NA	NA
**7**	Shinji Itoh	7.5 (0.1–45.9)/10.1 (0.1–28.3)	NA	NA	The Visera Elite II (Olympus, Tokyo, Japan) or IMAGE1 S Camera Systems (KARL STORZ, El Segundo, CA, USA)
**8**	Wang Jianxi	NA	0.125-0.25/2.5	Peripheral veins and portal veins	NA
**9**	Fusheng Liu	NA	0.025-0.25/0.25-0.5	Peripheral veins and portal veins	The Canadian Pinpoint Novadaq laparoscopic fluorescence imaging system
**10**	Yi Zhou	NA	0.025-0.125/0.125	Peripheral veins and portal veins	NA
**11**	Zhu Wen	7.65 (6.45–9.58)/6.85 (5.78–8.60)	2.5	Peripheral vein	NA

ICG, indocyanine green; ICG R15, Retention rate of indocyanine green at 15 minutes; NA, not applicable.

### 3.2 Surgery duration (min)

**Figure 2:**
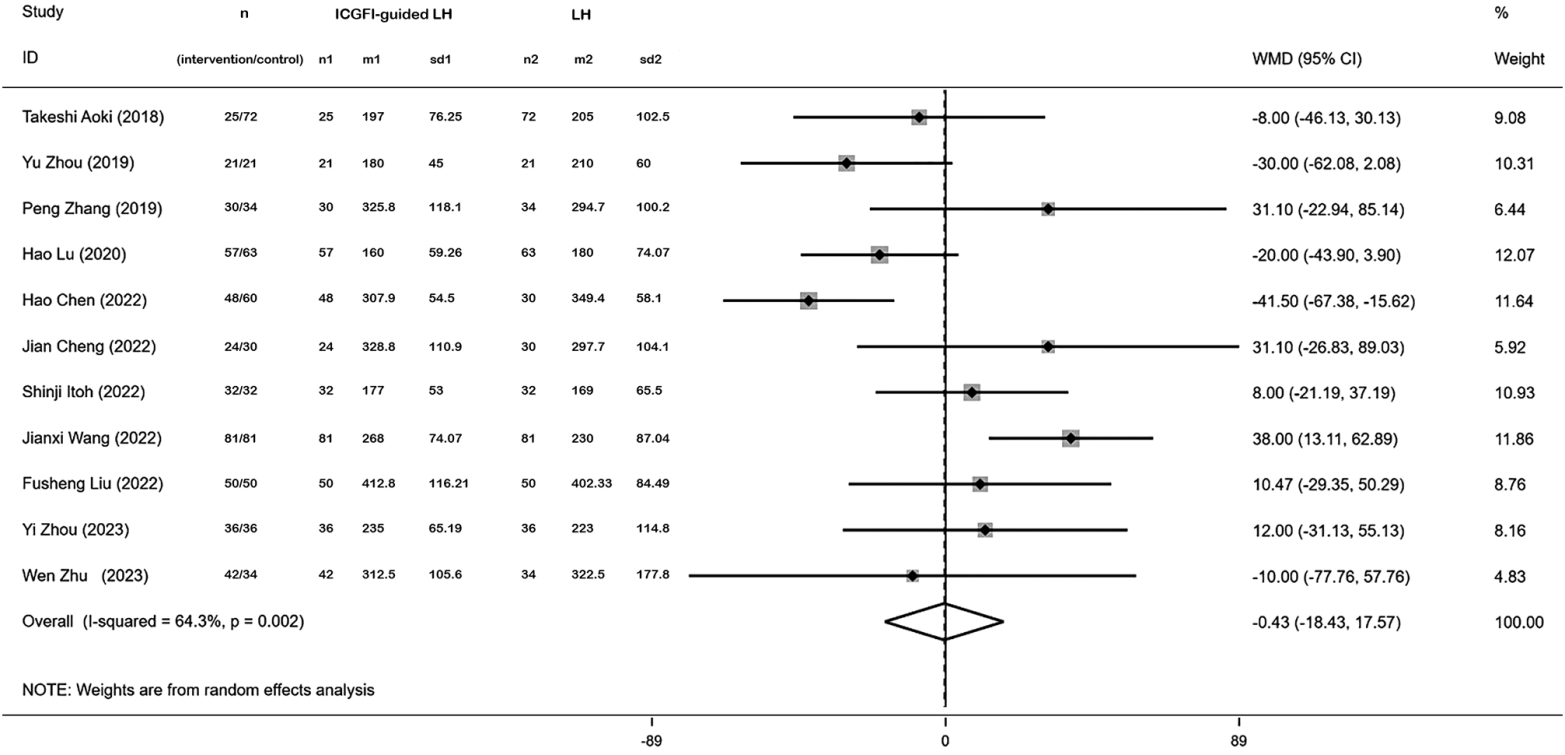
Forest plots of operation time.

Among the studies, we included five provided data on surgery duration. The heterogeneity assessment revealed great heterogeneity among these studies (*I^2 =^* 64.3%, *P* = 0.002). Consequently, we opted for a random-effects model for data analysis. The meta-analysis of the five studies revealed no significant difference regarding surgery duration between the two groups (WMD: −0.43, 95% CI: −18.43, 17.57, *P* = 0.963) (**Figure 2**).

### 3.3 R0 resection and minimal margin width (mm)

**Figure 3:**
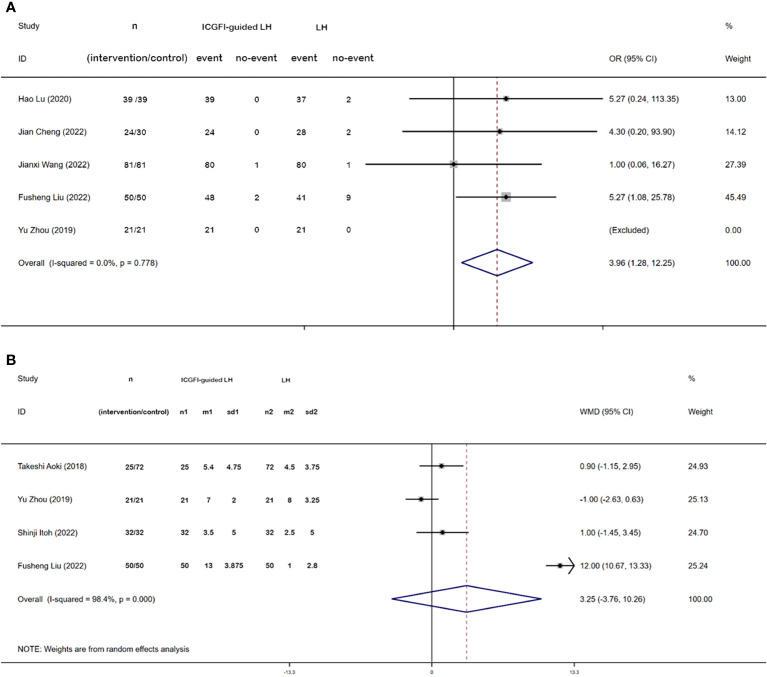
Forest plots of R0 resection **(A)** and Minimal margin width **(B)**.

Five studies contributed to data regarding the R0 resection rate. We employed a fixed-effects model for data synthesis given minimal heterogeneity across the five studies (*I^2 =^* 0.00%, *P* = 0.778). The meta-analysis revealed a significantly higher R0 resection rate in the ICGFI-guided LH group (OR: 3.96, 95% CI: 1.28, 12.25, *P* = 0.017) (**Figure 3A**). Four studies provided information on the minimal margin width. Utilizing a random-effects model, we found no statistically significant differences between the two groups (WMD = 3.25, 95% CI = −3.76, 10.26, *I^2 =^* 98.4%, P = 0.000) (**Figure 3B**).

### 3.4 Blood transfusion during operation

**Figure 4:**
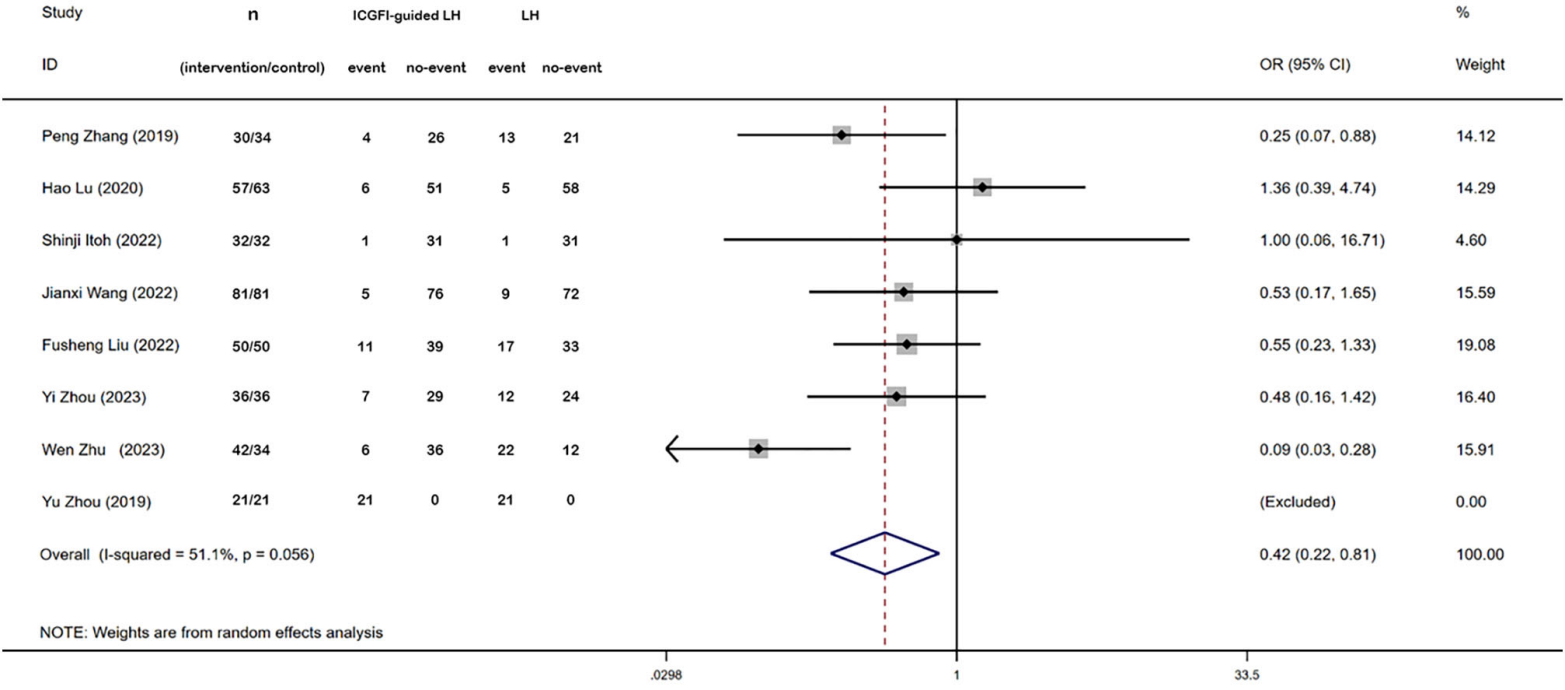
Forest plots of blood transfusion.

Blood transfusion was reported in seven studies. Heterogeneity testing unveiled great heterogeneity among these studies (*I^2 =^* 51.1%, *P* = 0.056), leading us to adopt a random-effects model. The meta-analysis demonstrated that patients undergoing ICGFI-guided LH exhibited a lower rate of blood transfusion (OR:0.42, 95% CI: 0.22, 0.81, *P* = 0.01) (**Figure 4**).

### 3.5 Postoperative hospital stay (day)

**Figure 5:**
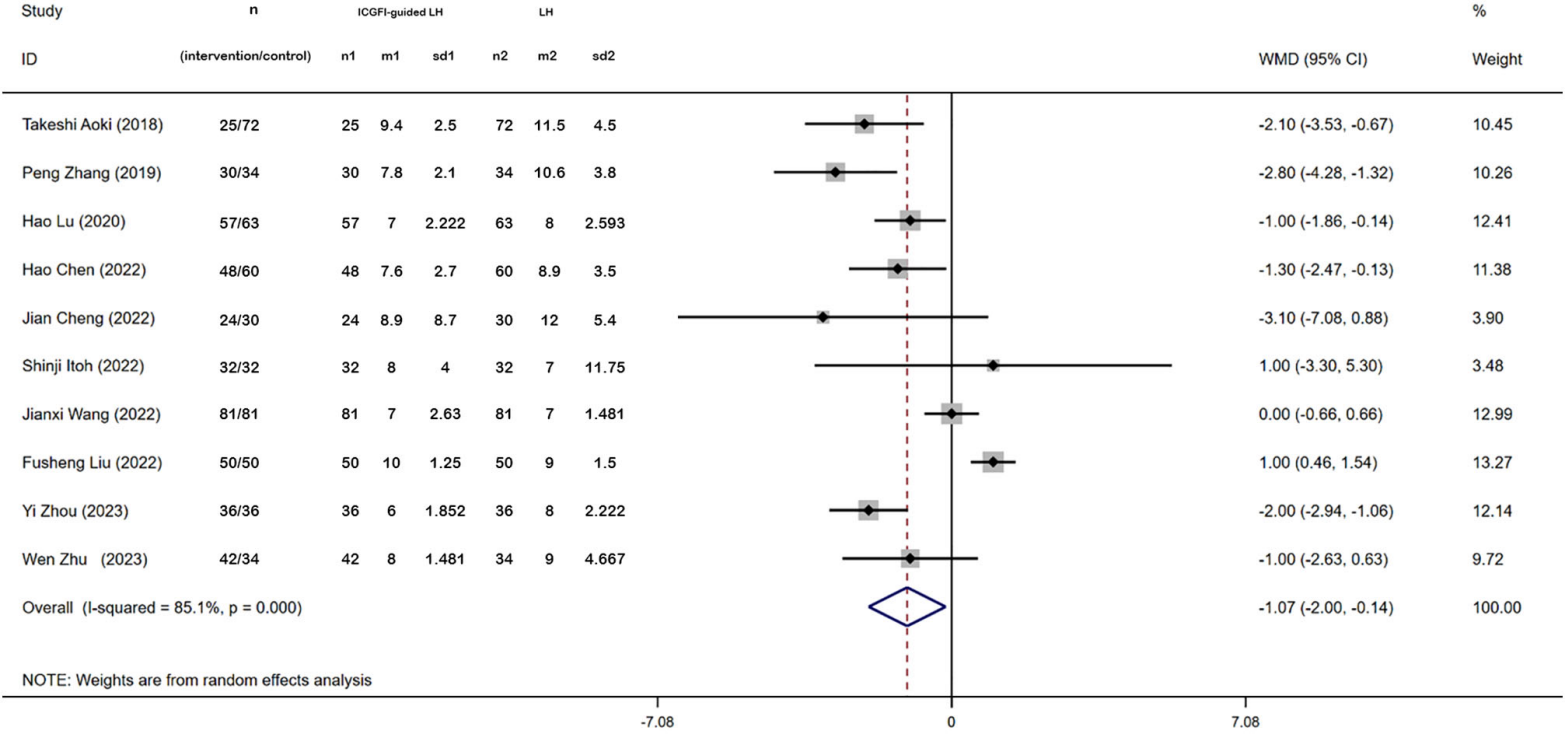
Forest plots of postoperative hospitalization.

Ten studies provided data on the length of postoperative hospital stay. Heterogeneity analysis indicated significant variability among these studies (*I^2 =^* 85.1%, *P* = 0.000), prompting the use of a random-effects model. The meta-analysis revealed that patients receiving ICGFI-guided LH experienced shorter postoperative hospital stay (WMD: −1.07, 95% CI: −2.00, −0.14, *P* = 0.023) (**Figure 5**).

### 3.6 Postoperative complications

This study assessed the risk of five postoperative complications, including the incidence of biliary fistula, hepatic failure, incision-related conditions, pleural effusion, and abdominal ascites. However, when compared to LH, there were no significant differences in the risk of postoperative complications between the two groups (Biliary Fistula: OR: 0.66, 95% CI: 0.27, 1.59, *I^2 =^* 0.000%, *P*=0.798; Hepatic Failure: OR:0.75, 95%CI: 0.20, 2.79, *I^2 =^* 37.7%, *P* = 0.205; Incision-related Conditions: OR:0.30, 95% CI: 0.06, 1.5, *I^2 =^* 0.00%, *P* = 0.981; Pleural Effusion: OR:1.20, 95% CI: 0.65, 2.23, *I^2 =^* 0.00%, *P* = 0.421; Abdominal Ascites: OR:0.90, 95% CI: 0.44, 1.86, *I^2 =^* 0.00%, *P* = 0.895) (**Supplementary Figures S1A–E**).

### 3.7 Publication bias and sensitivity analysis

To evaluate potential publication bias concerning operation time, we conducted both Begg’s test and Egger’s test. The funnel plot displayed a symmetrical distribution, suggesting the absence of publication bias (Begg’s test: P = 0.35; Egger’s test: P = 0.46) (**Supplementary Figure S2**). Additionally, we conducted a sensitivity analysis, where data were divided into sequences and then tested the sensitivity of the results to changes in those sequences, which confirmed the robustness of the overall results of our meta-analysis.

## 4 Discussion

This meta-analysis compared the efficacy of fluorescent laparoscopic hepatectomy with conventional laparoscopic hepatectomy in the treatment of liver tumors. Our analysis results demonstrated that ICGFI-guided LH, through precise and effective tumor removal, exhibited a remarkable ability to reduce the necessity for blood transfusions and it significantly shortened the length of postoperative hospital stay. It is noteworthy that no significant differences emerged between the two groups regarding minimal margin width and postoperative complications.

Our study elucidated that ICGFI-guided LH did not compromise the minimum resection margin width while concurrently enhancing the R0 resection rate, effectively accomplishing precise hepatectomy. Currently, patients with primary liver cancer can achieve favorable outcomes through precise surgical intervention (36). However, given the heterogeneity of tumor differentiation and the propensity for intrahepatic metastasis, mere lesion removal may result in incomplete eradication and subsequent disease recurrence (37). Despite the undeniable advantages of endoscopic procedures compared to open surgery, the decreased sensitivity of robotic arms may compromise the completeness of lesion resection, posing challenges in selecting the most suitable surgical methods (38). In recent years, advancements in visualization technology have brought to the fore the exceptional suitability of ICG as a marker for hepatocellular carcinomatous lesions. Of particular significance is its remarkable capacity to distinctly delineate lesion margins, thereby enabling complete removal through endoscopic surgery (39). The present study aimed to assess the effectiveness and safety of ICGFI in laparoscopic hepatectomy, drawing upon a comprehensive dataset gleaned from both domestic and international research. Our team hoped to provide robust evidence-based medical support for the integration of ICGFI into laparoscopic hepatectomy.

Our findings underscore the favorable efficacy and safety of ICGFI imaging in assisting laparoscopic liver tumor resection. These remarkable benefits primarily emanate from its ability to curtail blood transfusion rates, shorten hospital stay, augment the R0 resection rate, and reduce postoperative complications. The advantages of ICGFI can be attributed to several key features: Firstly, it provides a clear demarcation line, serving as a crucial reference point for endoscopic surgical resection. The administration of ICG following intraoperative blood vessel ligation within the target segment enables precise visualization of the intended area, effectively averting inadvertent incisions in adjacent segments during surgery (39). Moreover, ICG administration following vascular occlusion facilitates accurate identification of connecting blood vessels between segments, thereby enhancing intraoperative vascular management and minimizing the risk of intraoperative bleeding (28). Secondly, ICG fluorescence staining reduces the need for frequent and extended intraoperative ultrasound during liver parenchymal section dissection (40). Furthermore, ICGFI facilitates achieving standard anatomic hepatectomy in laparoscopic procedures, subsequently expediting postoperative liver function recovery and reducing the occurrence of postoperative complications, aligning with the principle of rapid rehabilitation (41). Finally, Indocyanine green fluorescence imaging allows the detection of superficial tumor lesions that are undetectable by preoperative imaging or intraoperative ultrasound, thereby increasing the detection rate and improving the accuracy of liver surgery and the efficacy of tumor treatment (42).

Furthermore, patients in the ICGFI-guided LH group experienced shorter postoperative hospital stay, contrary to the opposing viewpoint held by another meta-analysis (43). The presence of conflicting results might be attributed to variations in time periods and number of studies included. This meta-analysis, characterized by its inclusion of the most recent articles available up to 2023, adhered to a rigorous literature selection process. Moreover, advancements in medical devices and increased proficiency in surgical techniques undoubtedly contributed to enhanced patient recovery and a subsequent reduction in postoperative hospital stay.

Additionally, our study unveiled no significant difference concerning the risk of postoperative complications between the two groups. Although previous studies (44–47) have suggested that laparoscopic hepatectomy might potentially impact diaphragm, bile duct, and hepatic ligament anatomy, leading to complications such as ascites, pleural effusion, and biliary fistula, our study did not reveal such distinctions between the two groups. Nevertheless, the reliability of our results may be somewhat compromised due to the limited number of original studies reporting postoperative complications. Therefore, we underscore the imperative need for future research endeavors to delve deeper into both short-term and long-term complications ensuing from ICGFI-guided LH vs. LH without the assistance of ICGF.

However, it is worth noting that this meta-analysis does have certain limitations. Firstly, the included studies were cohort studies, which may introduce some degree of selection bias due to the absence of data from RCTs. Secondly, in some of the results, significant heterogeneity was observed, and despite conducting sensitivity analysis and subgroup analyses, pinpointing the specific sources of this heterogeneity proved elusive. Thirdly, our study did not detect any publication bias using the funnel plot and Egger’s test, the limited number of studies and small sample size could potentially impact the stability of the analysis results. Despite these limitations, we conducted the latest and largest meta-analysis of cohort studies to evaluate the role of ICGFI in laparoscopic hepatectomy for hepatic tumors.

## 5 Conclusion

In conclusion, the integration of ICGFI into laparoscopic hepatectomy for hepatic tumors offers significant benefits, including a significant reduction in blood transfusion rates, shorter postoperative hospital stay, enhanced R0 resection rates, and a comparable risk of postoperative complications when compared to traditional laparoscopic hepatectomy. Nonetheless, further large-scale, multicenter, double-blind RCTs are required to furnish more robust evidence and confirm the safety and effectiveness of ICGFI IN laparoscopic hepatectomy for hepatic tumors.

## Data availability statement

The original contributions presented in the study are included in the article/**Supplementary Material**. Further inquiries can be directed to the corresponding author.

## Author contributions

KZ: Data curation, Formal analysis, Investigation, Methodology, Project administration, Software, Validation, Visualization, Writing – original draft, Writing – review & editing. SZ: Data curation, Methodology, Writing – review & editing. LD: Conceptualization, Project administration, Supervision, Writing – original draft. EL: Data curation, Formal analysis, Methodology, Writing – original draft. HD: Conceptualization, Project administration, Supervision, Validation, Visualization, Writing – review & editing. FM: Conceptualization, Project administration, Supervision, Validation, Visualization, Writing – review & editing. YS: Conceptualization, Project administration, Supervision, Writing – review & editing. YL: Conceptualization, Methodology, Software, Supervision, Validation, Visualization, Writing – review & editing.

## Supplementary material

Supplementary DATA SHEET 1 Search strategy.

Supplementary DATA SHEET 2 Newcastle-Ottawa Scale.
