# Supplementary material for: Safety and effectiveness of indocyanine green fluorescence imaging-guided laparoscopic hepatectomy for hepatic tumor: a systematic review and meta-analysis
Source: Front Oncol. 2024 Jan 3;13:1309593. doi: 10.3389/fonc.2023.1309593 (PMC10791760; doi:10.3389/fonc.2023.1309593)
Supplement: Supplementary DATA SHEET 1 — Search strategy. [file DataSheet_1.docx]

**Safety and effectiveness of** **indocyanine green fluorescence imaging-guided laparoscopic hepatectomy for hepatic tumor: A systematic review and meta-analysis**

**Running title:** Indocyanine green fluorescence in laparoscopic hepatectomy

**Kan Zhou^1^, Shumin Zhou^2^, Lei Du^1^, Erpeng Liu^3^, Hao Dong^1^, Fuping Ma^1^, Yali Sun^1^, Ying Li^1, *^**

***Corresponding author:** Ying LiEmail: 1263599603@qq.com

**Search strategy in PubMed**

("indocyanine green"[MeSH Terms] OR ("cardio green"[Title/Abstract] OR "cardiogreen"[Title/Abstract] OR "cardio green"[Title/Abstract] OR "diagnogreen"[Title/Abstract] OR "fox green"[Title/Abstract] OR "green indocyanine"[Title/Abstract] OR (("infect control"[Journal] OR "infect chemother"[Journal] OR "ic"[All Fields]) AND "2000"[Title/Abstract]) OR "ic green"[Title/Abstract] OR "icg"[Title/Abstract] OR "ic green"[Title/Abstract] OR "indocyanide green"[Title/Abstract] OR "indocyanin green"[Title/Abstract] OR "indocyanin green test"[Title/Abstract] OR "indocyanine"[Title/Abstract] OR "indocyanine green test"[Title/Abstract] OR (("indocyanin"[All Fields] OR "indocyanine"[All Fields] OR "indocyanines"[All Fields]) AND "test"[Title/Abstract]) OR (("spy"[All Fields] AND ("agent"[All Fields] OR "agents"[All Fields])) AND "green"[Title/Abstract]) OR (("spy"[All Fields] AND ("agent"[All Fields] OR "agents"[All Fields])) AND "green kit"[Title/Abstract]) OR (("tricarbocyanine"[All Fields] OR "tricarbocyanines"[All Fields]) AND "ii"[Title/Abstract]) OR "vofaverdin"[Title/Abstract] OR "wofaverdin"[Title/Abstract])) AND ("liver neoplasms"[MeSH Terms] OR ("cancer of liver"[Title/Abstract] OR "cancer of the liver"[Title/Abstract] OR "cancer hepatic"[Title/Abstract] OR "cancer hepatocellular"[Title/Abstract] OR "cancer liver"[Title/Abstract] OR "cancers hepatic"[Title/Abstract] OR "cancers hepatocellular"[Title/Abstract] OR "cancers liver"[Title/Abstract] OR "hepatic cancer"[Title/Abstract] OR "hepatic cancers"[Title/Abstract] OR "hepatic neoplasm"[Title/Abstract] OR "hepatic neoplasms"[Title/Abstract] OR "hepatocellular cancer"[Title/Abstract] OR "hepatocellular cancers"[Title/Abstract] OR "liver cancer"[Title/Abstract] OR "liver cancers"[Title/Abstract] OR "liver neoplasm"[Title/Abstract] OR "neoplasm hepatic"[Title/Abstract] OR "neoplasm liver"[Title/Abstract] OR "neoplasms hepatic"[Title/Abstract] OR "neoplasms liver"[Title/Abstract]))

**Search strategy in Cochrane library**

#1 (‘Cardio Green’ OR ‘Cardiogreen’ OR ‘Cardio-Green’ OR ‘diagnogreen’ OR ‘fox green’ OR ‘Green, Indocyanine’ OR ‘ic 2000’ OR ‘ic green’ OR ‘ic2000’ OR ‘icg’ OR ‘ic-green’ OR ‘indocyanide green’ OR ‘indocyanin green’ OR ‘indocyanin green test’ OR ‘indocyanine’ OR ‘indocyanine green test’ OR ‘indocyanine test’ OR ‘spy agent green’ OR ‘spy agent green kit’ OR ‘tricarbocyanine ii’ OR ‘Ujoveridin’ OR ‘Vofaverdin’ OR ‘Wofaverdin’):ti,ab,kw

#2 MeSH descriptor: [Indocyanine Green] explode all trees

#3 (‘Cancer of Liver’ OR ‘Cancer of the Liver’ OR ‘Cancer, Hepatic’ OR ‘Cancer, Hepatocellular’ OR ‘Cancer, Liver’ OR ‘Cancers, Hepatic’ OR ‘Cancers, Hepatocellular’ OR ‘Cancers, Liver’ OR ‘Hepatic Cancer’ OR ‘Hepatic Cancers’ OR ‘Hepatic Neoplasm’ OR ‘Hepatic Neoplasms’ OR ‘Hepatocellular Cancer’ OR ‘Hepatocellular Cancers’ OR ‘Liver Cancer’ OR ‘Liver Cancers’ OR ‘Liver Neoplasm’ OR ‘Neoplasm, Hepatic’ OR ‘Neoplasm, Liver’ OR ‘Neoplasms, Hepatic’ OR ‘Neoplasms, Liver’ ):ti,ab,kw

#4 MeSH descriptor: [Liver Neoplasms] explode all trees

#5 (#1 OR #2) AND (#3 OR #4)

**Search strategy in Web of Science**

#1 (TS=(liver neoplasms)) OR TS=("Cancer of Liver" OR "Cancer of the Liver" OR "cancer hepatic" OR "cancer hepatocellular" OR "cancer liver" OR "cancers hepatic" OR "cancers hepatocellular" OR "cancers liver" OR "Hepatic Cancer" OR "Hepatic Cancers" OR "Hepatic Neoplasm" OR "Hepatic Neoplasms" OR "Hepatocellular Cancer" OR "Hepatocellular Cancers" OR "Liver Cancer" OR "Liver Cancers" OR "Liver Neoplasm" OR "neoplasm hepatic" OR "neoplasm liver" OR "neoplasms hepatic" OR "neoplasms liver")

#2 (TS=(indocyanine green)) OR TS=("4 [2 [7 [1, 1 dimethyl 3 (4 sulfonatobutyl) benzo [e] indol 3 ium 2 yl] hepta 2, 4, 6 trienylidene] 1, 1 dimethylbenzo [e] indol 3 yl] butane 1 sulfonate sodium" OR "Cardio Green" OR "Cardiogreen" OR "Cardio-Green" OR "diagnogreen" OR "fox green" OR "Green, Indocyanine" OR "ic 2000" OR "ic green" OR "ic2000" OR "icg" OR "ic-green" OR "indocyanide green" OR "indocyanin green" OR "indocyanin green test" OR "indocyanine" OR "indocyanine green test" OR "indocyanine test" OR "spy agent green" OR "spy agent green kit" OR "tricarbocyanine ii" OR "Ujoveridin" OR "Vofaverdin" OR "Wofaverdin")

#1 AND #2

**Search strategy in Embase**

#1 'cardio green':ti,ab,kw OR 'cardiogreen':ti,ab,kw OR 'cardio-green':ti,ab,kw OR 'diagnogreen':ti,ab,kw OR 'fox green':ti,ab,kw OR 'green, indocyanine':ti,ab,kw OR 'ic 2000':ti,ab,kw OR 'ic green':ti,ab,kw OR 'ic2000':ti,ab,kw OR 'icg':ti,ab,kw OR 'ic-green':ti,ab,kw OR 'indocyanide green':ti,ab,kw OR 'indocyanin green':ti,ab,kw OR 'indocyanin green test':ti,ab,kw OR 'indocyanine':ti,ab,kw OR 'indocyanine green test':ti,ab,kw OR 'indocyanine test':ti,ab,kw OR 'spy agent green':ti,ab,kw OR 'spy agent green kit':ti,ab,kw OR 'tricarbocyanine ii':ti,ab,kw OR 'ujoveridin':ti,ab,kw OR 'vofaverdin':ti,ab,kw OR 'wofaverdin':ti,ab,kw

#2 'indocyanine green'/exp

#3 'cancer of liver':ti,ab,kw OR 'cancer of the liver':ti,ab,kw OR 'cancer, hepatic':ti,ab,kw OR 'cancer, hepatocellular':ti,ab,kw OR 'cancer, liver':ti,ab,kw OR 'cancers, hepatic':ti,ab,kw OR 'cancers, hepatocellular':ti,ab,kw OR 'cancers, liver':ti,ab,kw OR 'hepatic cancer':ti,ab,kw OR 'hepatic cancers':ti,ab,kw OR 'hepatic neoplasm':ti,ab,kw OR 'hepatic neoplasms':ti,ab,kw OR 'hepatocellular cancer':ti,ab,kw OR 'hepatocellular cancers':ti,ab,kw OR 'liver cancer':ti,ab,kw OR 'liver cancers':ti,ab,kw OR 'liver neoplasm':ti,ab,kw OR 'neoplasm, hepatic':ti,ab,kw OR 'neoplasm, liver':ti,ab,kw OR 'neoplasms, hepatic':ti,ab,kw OR 'neoplasms, liver':ti,ab,kw

#4 'liver tumor'/exp

#5 (#1 OR #2) AND (#3 OR #4)
