# Supplementary material for: Safety and effectiveness of indocyanine green fluorescence imaging-guided laparoscopic hepatectomy for hepatic tumor: a systematic review and meta-analysis
Source: Front Oncol. 2024 Jan 3;13:1309593. doi: 10.3389/fonc.2023.1309593 (PMC10791760; doi:10.3389/fonc.2023.1309593)
Supplement: Supplementary DATA SHEET 2 — Newcastle-Ottawa Scale. [file DataSheet_2.docx]

**Supplementary material 2** Newcastle Ottawa Scale of included studies (n=11)

| study | year | Selection | | | | Comparability | Outcome | | | Total Points |
| --- | --- | --- | --- | --- | --- | --- | --- | --- | --- | --- |
|  |  | ① | ② | ③ | ④ | ⑤ | ⑥ | ⑦ | ⑧ |  |
| Takeshi Aoki | 2018 | 1 | 1 | 1 | 1 | 1 | 1 | 1 | 1 | 8 |
| Yu Zhou | 2019 | 1 | 1 | 1 | 1 | 1 | 1 | 1 | 1 | 8 |
| Peng Zhang | 2019 | 1 | 1 | 1 | 1 | 0 | 1 | 1 | 1 | 7 |
| Hao LU | 2020 | 1 | 1 | 1 | 1 | 1 | 1 | 1 | 1 | 8 |
| Hao Chen | 2022 | 1 | 1 | 1 | 1 | 1 | 1 | 0 | 1 | 7 |
| Jian Cheng | 2022 | 1 | 1 | 1 | 1 | 1 | 1 | 1 | 1 | 8 |
| Shinji Itoh | 2022 | 1 | 1 | 1 | 1 | 1 | 1 | 1 | 1 | 8 |
| Wang Jianxi | 2022 | 1 | 1 | 1 | 1 | 1 | 1 | 1 | 1 | 8 |
| Fusheng Liu | 2022 | 1 | 1 | 1 | 1 | 1 | 1 | 1 | 1 | 8 |
| Yi Zhou | 2023 | 1 | 1 | 1 | 1 | 1 | 1 | 1 | 1 | 8 |
| Zhu Wen | 2023 | 1 | 1 | 1 | 1 | 1 | 1 | 0 | 1 | 7 |

Abbreviations: ①Representativeness of the exposed cohort ②Selection of the non exposed cohort ③Ascertainment of exposure

④Demonstration that outcome of interest was not present at start of study ⑤Comparability of cohorts on the basis of the design or analysis

⑥Assessment of outcome ⑦Was follow-up long enough for outcomes to occur ⑧Adequacy of follow up of cohorts
