# Supplementary material for: Safety and effectiveness of indocyanine green fluorescence imaging-guided laparoscopic hepatectomy for hepatic tumor: a systematic review and meta-analysis
Source: Front Oncol. 2024 Jan 3;13:1309593. doi: 10.3389/fonc.2023.1309593 (PMC10791760; doi:10.3389/fonc.2023.1309593)
Supplement: Supplementary file 3 [file DataSheet_3.docx]

**Safety and effectiveness of** **indocyanine green fluorescence imaging-guided laparoscopic hepatectomy for hepatic tumor: A systematic review and meta-analysis**

**Running title:** Indocyanine green fluorescence in laparoscopic hepatectomy

**Kan Zhou^1^, Shumin Zhou^2^, Lei Du^1^, Erpeng Liu^3^, Hao Dong^1^, Fuping Ma^1^, Yali Sun^1^, Ying Li^1, *^**

***Corresponding author:** Ying LiEmail: 1263599603@qq.com


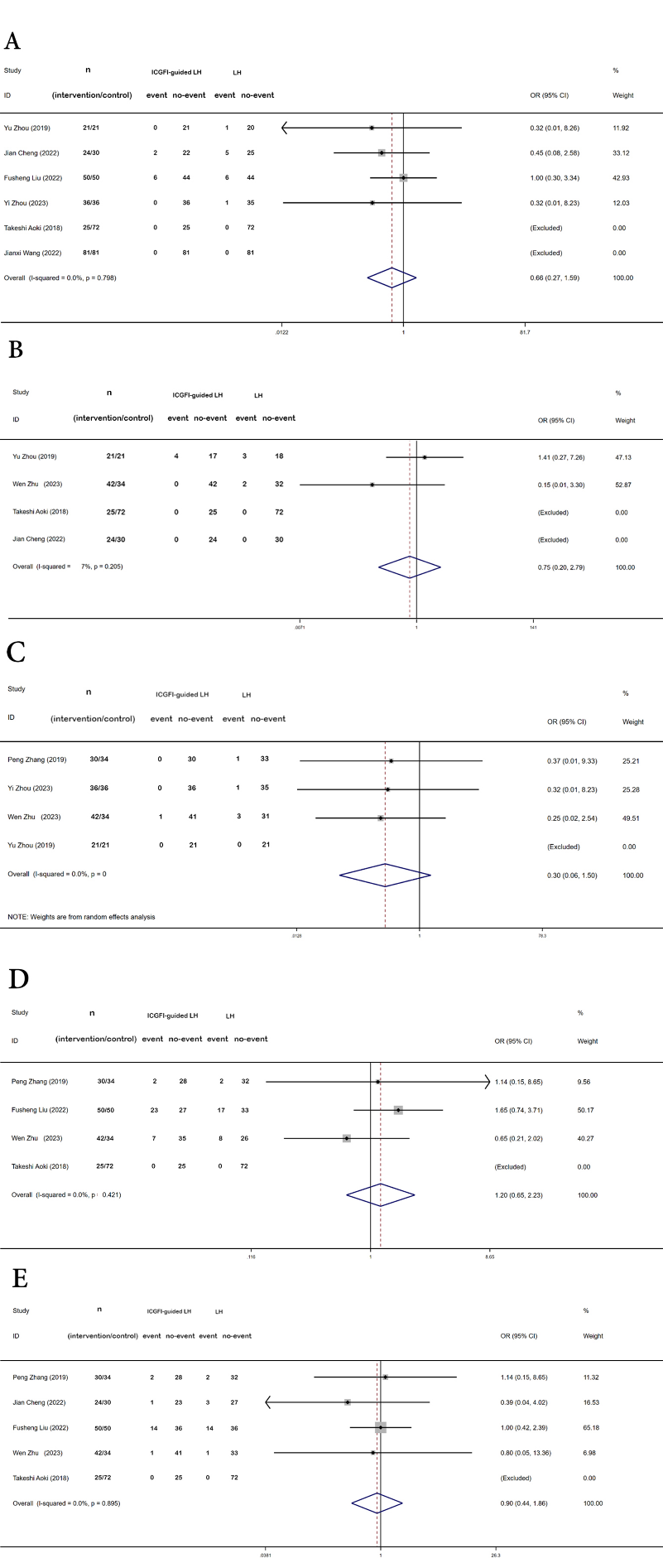


**Figure S1** Forest plots of biliary fistula(A), hepatic failure(B), incision(C), pleural effusion(D) and abdominal ascites(E)


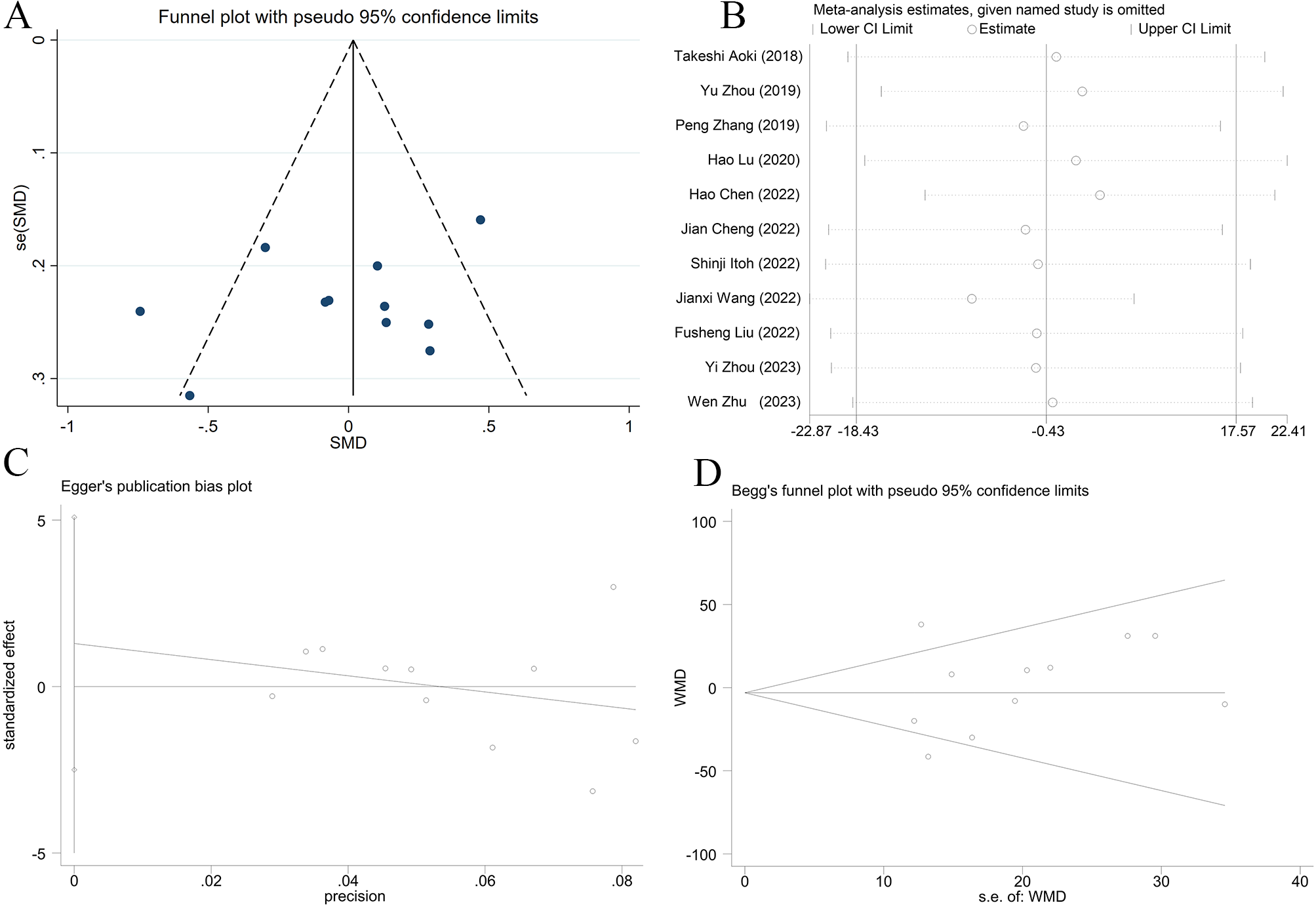


**Figure S2** Funnel plot of the meta-analysis, egger’s test and begg’s test
